# Prevalence, interreader agreement, and prognostic value of high-grade and relevant strictures in individuals with primary sclerosing cholangitis

**DOI:** 10.1007/s00330-026-12426-6

**Published:** 2026-03-13

**Authors:** Stefan Hamma, Annika Bergquist, Mats Andersson, Christina Villard, Aristeidis Grigoriadis

**Affiliations:** 1 Division of Radiology, Department of Clinical Science, Intervention and Technology (CLINTEC), Karolinska Institutet, Stockholm, Sweden; 2 Department of Radiology, Karolinska University Hospital, Stockholm, Sweden; 3 Unit of Gastroenterology and Nutrition (GUT), Department of Medicine Huddinge (MedH), Karolinska Institutet, Stockholm, Sweden; 4 Division of Hepatology, Department of Upper GI Disease, Karolinska University Hospital, Stockholm, Sweden; 5 Department of Transplantation Surgery, Karolinska University Hospital, Stockholm, Sweden

**Keywords:** Cholangitis (sclerosing), Constriction (pathologic), Prognosis, Magnetic resonance imaging, Observer variation

## Abstract

**Objectives:**

High-grade and relevant strictures have recently been introduced in clinical guidelines for primary sclerosing cholangitis (PSC). However, the definition of relevant strictures differs between the two liver associations (AASLD, EASL). We aim to assess the prevalence, the agreement of identification of extrahepatic, high-grade, and relevant strictures, and their association with outcomes in PSC.

**Materials and methods:**

In this retrospective single-center study, three radiologists, independently and in consensus, assessed MRCPs of 170 PSC individuals for the presence of extrahepatic and high-grade strictures. Interreader agreement was calculated with Fleiss kappa. Association of extrahepatic, high-grade, and relevant strictures with outcomes (hepatobiliary malignancy, liver transplantation, liver-related death) was assessed with Cox-regression, and outcome-free survival estimates with Kaplan–Meier.

**Results:**

Median age was 40 years, and 62% were males. One hundred-seven (63%) individuals had high-grade strictures, 49 (29%), and 53 (31%) had relevant strictures according to EASL and AASLD, respectively. During the median follow-up of 10.3 years, 50 individuals developed outcomes (liver transplantation = 37, liver-related death = 5, hepatobiliary malignancy = 8). Agreement for high-grade strictures was fair (k = 0.31). All strictures types were associated with worse prognosis in the univariate and multivariate analysis with extrahepatic strictures having hazard ratio (HR) = 3.34 (95% CI: 1.42–7.86), high-grade strictures HR = 2.00 (95% CI: 1.02–3.90), relevant strictures according to EASL and AASLD had HR = 2.43 (95% CI: 1.34–4.42) and HR = 2.89 (95% CI:1.57–5.32), respectively, after adjusting for Mayo Risk Score.

**Conclusion:**

Prevalence of high-grade strictures is high, but the agreement of their identification is unsatisfactory. High-grade and relevant strictures, regardless of their definition (EASL or AASLD), were associated with a worse prognosis.

**Key Points:**

***Question*** *What is the prevalence and the interreader agreement of high-grade and relevant strictures? Are they associated with a worse prognosis in PSC?*

***Findings*** *High-grade and relevant strictures are relatively common in PSC and are associated with a worse prognosis; however, the agreement of their identification is unsatisfactory*.

***Clinical relevance*** *High-grade and relevant strictures have a role in clinical practice; however, the low interreader agreement of the interpretation of MRCP of individuals with PSC remains an unmet challenge*.

**Graphical Abstract:**

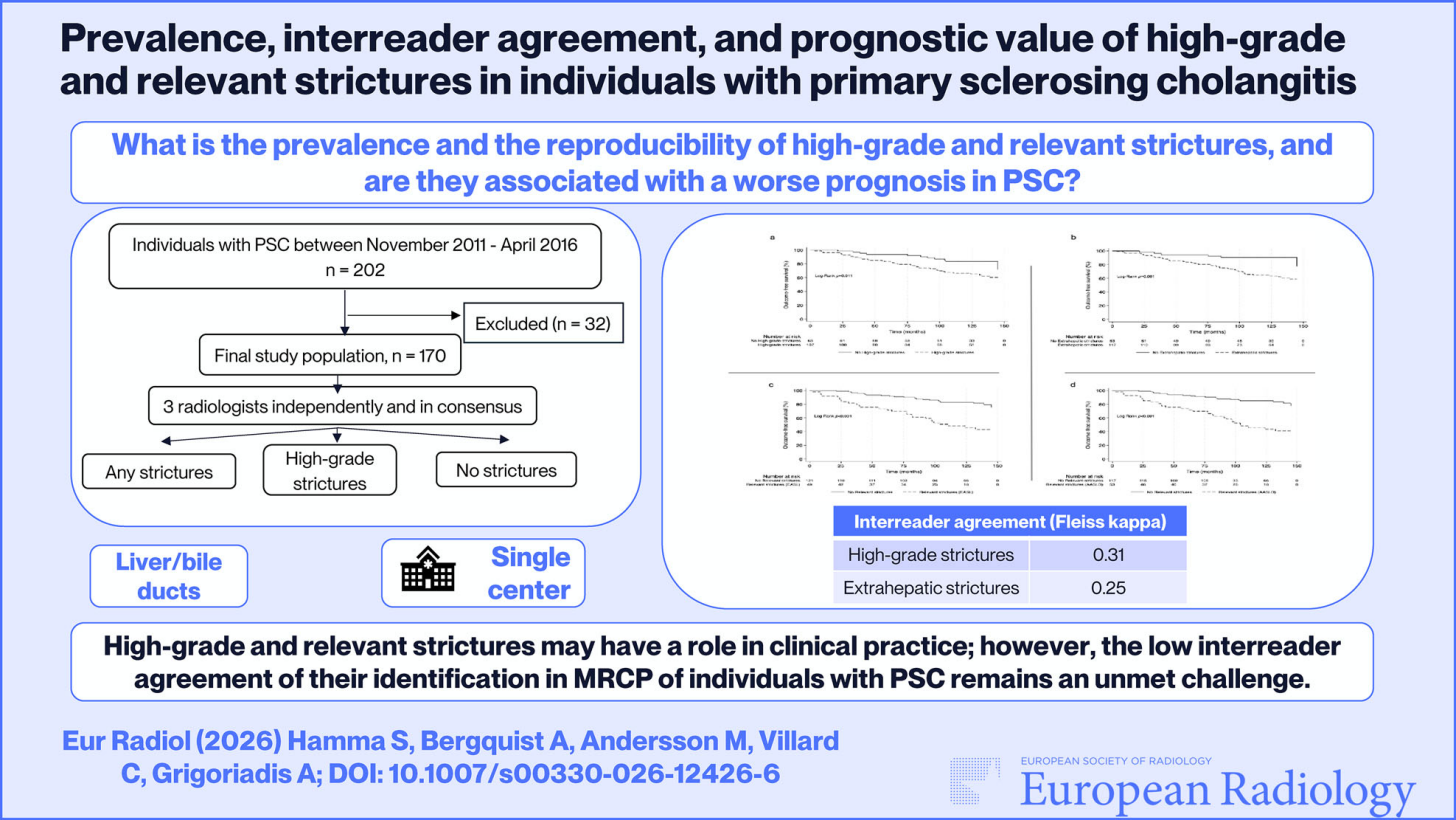

## Introduction

Primary sclerosing cholangitis (PSC) is a chronic inflammatory liver and biliary disease of unknown etiology, often leading to biliary cirrhosis. It is associated with an increased risk for the development of cholangiocarcinoma (CCA) [1]. The disease is characterized by peribiliary fibrosis leading to the formation of biliary strictures. The term dominant stricture (DS) has been the main descriptive term for clinically significant strictures in PSC since its introduction [2], and it has been associated with a poorer prognosis [3] and an increased risk for CCA [4]. DS was defined in endoscopic retrograde cholangiopancreatography (ERCP) as a biliary stricture with a diameter of the common bile/hepatic duct ≤ 1.5 mm and/or diameter of hepatic ducts within 2 cm from the primary biliary confluence ≤ 1 mm. DS can only be used in ERCP; nevertheless, it has been loosely applied in clinical practice and the evaluation of MRCP, occasionally leading to confusion and controversy.

The international PSC Study Group (IPSCSG) redefined DS by combining MRCP with clinical data and introduced the term probable DS [5]. The European Association for the Study of the Liver (EASL) and the American Association for the Study of Liver Diseases (AASLD), however, later agreed on abandoning the term DS at MRCP and instead adopted the more MRCP-specific term “high-grade stricture” [6–8]. High-grade strictures are defined as strictures that cause more than 75% reduction in the lumen of the common bile duct or hepatic ducts [6, 8]. In addition, a new term called “relevant stricture” was proposed to describe clinically significant extrahepatic strictures. The definition of a relevant stricture differs slightly between the two liver associations. EASL uses the term “high-grade stricture,” while AASLD uses “any stricture” in the extrahepatic biliary tree in conjunction with signs or symptoms of bacterial cholangitis and/or obstructive cholestasis. DS and high-grade/relevant strictures are not identical [7], and the results of earlier studies on DS cannot be safely extrapolated to strictures detected at MRCP. Therefore, the significance of high-grade and relevant strictures for predicting outcomes in PSC remains unknown. Moreover, despite the introduction of high-grade strictures in MRCP some years ago, the reproducibility of their identification has not been adequately studied.

The aims of this study are to assess the prevalence and the interreader agreement in identifying extrahepatic strictures and high-grade strictures, and to investigate the prognostic value of all types of extrahepatic strictures in individuals with large duct PSC.

## Materials and methods

### Study population

This retrospective cohort study was conducted in accordance with the Declarations of Helsinki and Istanbul. Informed written consent was obtained from all participants at inclusion in the SUPRIM study. The Swedish ethical review authority approved the study (2011/824-31/2, 2018/1111-32, and 2018/1494-31/3).

**Fig. 1:**
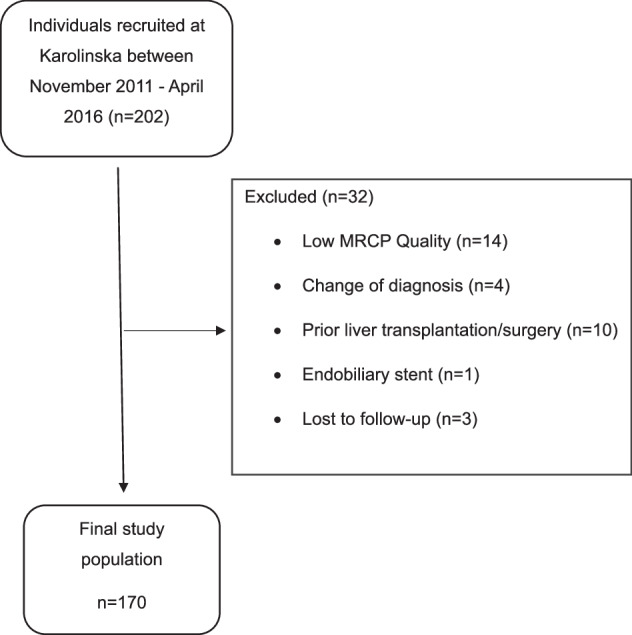
Flowchart of the study population

We included consecutive adult individuals with large duct PSC recruited in the SUPRIM cohort at Karolinska University Hospital in Stockholm, Sweden, between 2011 and 2016 [9, 10]. Participants underwent MRI/MRCP at inclusion and then annually according to a dedicated MRI/MRCP protocol (Table S1). The MRI/MRCP examinations closest to inclusion were retrieved for evaluation in the present study. All examinations were part of the surveillance protocol and, thus, not performed due to a change or worsening of symptoms or liver tests. Demographic and clinical-laboratory data, together with clinical scores such as MELD and MRS scores at inclusion, were collected and are presented in Table 1. Outcome data until March 2024 were collected. Exclusion criteria were insufficient MRCP quality, the presence of biliary stents at the time of MRCP, liver surgery, or the development of any of the study outcomes before the MRCP date. Figure 1 presents the flowchart of the study population.

**Table 1:** Demographic, laboratory, and outcome data of the study population

	Study population	Normal range
Number of patients	170	-
Male/female	106/64	-
Age in years (at MRI date)	40 (19–81)	-
IBD, *n* (%)		-
Ulcerative colitis	98 (58)	-
Crohn’s disease	36 (21)	-
Indeterminate colitis	3 (2)	-
No IBD	33 (19)	-
AIH	21/156	-
UDCA	111/156	-
Bilirubin (μmol/L)	11 (2–71)	< 26
Bilirubin > 50 μmol/L (*n*)	2/170	-
ALAT (μkat/L)	0.8 (0.1–11.8)	< 1.1
ASAT (μkat/L)	0.67 (0.21–7.8)	< 0.76
GT (μkat/L)	2.2 (0.1–55.8)	< 1.4
ALP (μkat/L)	2.1 (0.6–14.7)	0.7–1.9
ALP > × 3 ULN (*n*)	24/170	-
Albumin (g/L)	39 (5.1–51)	36–48
CA 19-9 (kE/L)	9.4 (0.3–154)	< 34
MELD score	6 (6–17)	-
Mayo risk score	−0.24 (−5.1 to 2.63)	-
Follow-up time from MRI (years)	10.3 (0.2–14)	-
Clinical events at follow-up, *n* (%)	50 (29)	-
Liver Tx	37 (22)	-
Liver-related death	5 (3)	-
Hepatobiliary cancer	8 (5)	-
CCA	4 (2)	-
HCC	2 (1)	-
Gallbladder cancer	2 (1)	-

All values expressed as medians (range or percentage in parentheses) unless otherwise indicated

*AIH* autoimmune hepatitis, *ALAT* alanine aminotransferase, *ALP* alkaline phosphatase, *ULN* upper limit normal, *ASAT* aspartate transaminase, *CCA* cholangiocarcinoma, *GT* glutamyl transferase, *HCC* hepatocellular carcinoma, *IBD* inflammatory bowel disease, *Liver Tx* liver transplantation, *MELD* model for end-stage liver disease, *MRI* magnetic resonance imaging, *UDCA* ursodeoxycholic acid

### Definitions

All types of strictures assessed were located in the extrahepatic biliary tree, including the common bile duct, common hepatic duct, right and left hepatic ducts. An extrahepatic stricture was defined as a reduction in the diameter of the lumen of the extrahepatic bile ducts. A high-grade stricture was defined as a stricture causing > 75% reduction of the diameter of the common bile duct or hepatic ducts, as defined by IPSCSG [7] and described in EASL and AASLD guidelines [6, 8]. Relevant strictures were defined as extrahepatic strictures (AASLD) or high-grade strictures (EASL) with signs or symptoms of obstructive cholestasis and/or bacterial cholangitis (bilirubin > 50 μmol/L, itch, × 3 times increase of ALP, cholangitis that needed medical treatment with antibiotics).

The study´s composite outcome included liver transplantation, development of hepatobiliary malignancy (CCA, gallbladder cancer, hepatocellular carcinoma), or liver-related death, whichever occurred first.

### Image acquisition

MRI/MRCP examinations were performed using 1.5-T scanners (SIEMENS Magnetom Aera or Avanto, SIEMENS Healthineers) with an 18-channel body coil, according to a standardized protocol as previously described by our group (Table S1) [11]. For this study, only 3D MRCP acquired in the coronal plane for the identification of strictures and axial T2-weighted images for anatomical correlation were utilized.

### Image evaluation

Three radiologists (S.H., A.G., M.A.) working at the radiology department at Karolinska University Hospital in Stockholm, Sweden, with 5, 12, and 32 years of experience in abdominal radiology, independently evaluated the images in a commercially available picture archiving and communication system (PACS) workstation. The readers were asked to record the presence or absence of both extrahepatic strictures and high-grade strictures in the extrahepatic biliary tree (as a categorical variable). No prior formal training sessions to identify the specific types of strictures evaluated in the study were conducted. All three radiologists were instructed to adhere to the definitions provided by EASL and AASLD [6, 8]. In addition, each reader documented the anatomical location of high-grade strictures using predefined categories: (1) no HGS, (2) common bile duct (CBD), (3) common hepatic duct (CHD), (4) right hepatic duct (RHD), (5) left hepatic duct (LHD), (6) primary confluence, or (7) multiple strictures. During their reading sessions, each radiologist assessed whether the image quality of each MRCP was sufficient for the assessment of the presence or absence of strictures and high-grade strictures. MRCPs inadequate for this assessment were excluded. The radiologists were free to perform maximum intensity projection (MIP) and multiplanar reformations from the 3D MRCP dataset. Cases of disagreement were resolved in an additional consensus reading. The readers were aware of the PSC diagnosis but were blinded to other clinical and radiological findings, including outcomes.

### Statistical analysis

The level of interreader agreement between the three radiologists in identifying extrahepatic and high-grade strictures was assessed using the Fleiss kappa [12, 13]. The kappa values were categorized as slight (0.0 < k < 0.2), fair (0.21 < k < 0.40), moderate (0.41 < k < 0.60), substantial (0.61 < k < 0.80), and excellent (k > 0.81) agreement. The association between all types of assessed strictures and scores with outcomes was evaluated using Cox proportional-hazards regression analysis. A univariate analysis was conducted initially, followed by the construction of multiple multivariate models. Each model included only one type of stricture along with the Mayo Risk Score (MRS). Outcome-free survival rates were assessed with Kaplan–Meier estimates and compared using the log-rank test. The consensus evaluation was used for survival analysis. A *p*-value less than 0.05 was considered statistically significant. All analyses were conducted using STATA, version 18 and 19.

## Results

### Clinical characteristics and outcomes

The final study population consisted of 170 individuals with large duct PSC. One hundred-six (62%) were males, and the median age at diagnosis was 40 years. One hundred thirty-seven (81%) had inflammatory bowel disease. The median time between MRCP and the last follow-up or outcome was 10.3 years (0.2–14 years). During the study period, 37 individuals underwent liver transplantation, eight developed hepatobiliary malignancy (CCA = 4, gallbladder cancer = 2, hepatocellular carcinoma = 2), and five died from liver-related causes. Seventy individuals (41%) had at least one sign or symptom of bacterial cholangitis or obstructive cholestasis according to the study definition. Results of liver function tests and scores are displayed in Table 1.

We additionally compared basic demographic and laboratory/clinical characteristics of the excluded patients to the final study population to evaluate potential attrition bias. The results are presented in Supplementary Table 6. Bilirubin was the only variable showing a statistically significant difference, with higher values among excluded patients; however, both medians remained within the normal range (11 vs. 19 μmol/L).

### Assessment of agreement

**Fig. 2:**
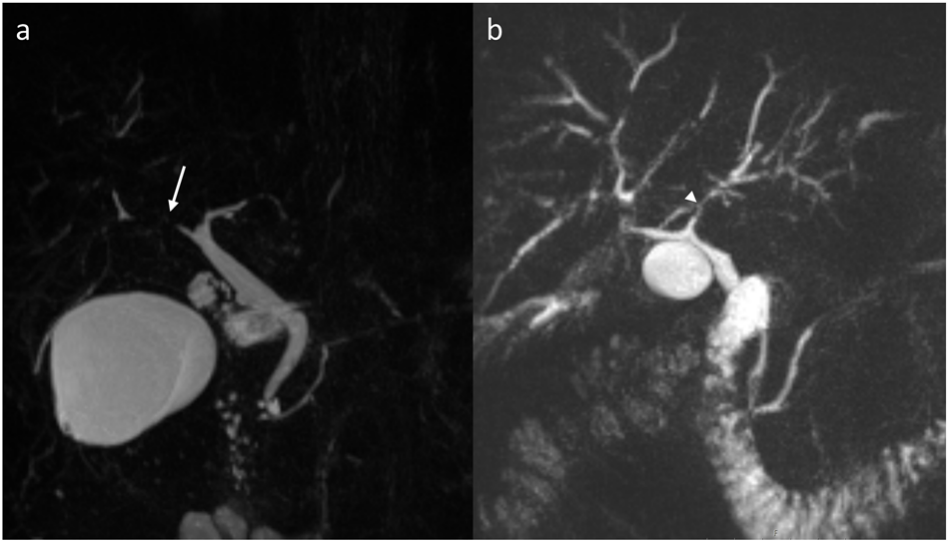
Cases of agreement and disagreement between readers in the evaluation of the presence of high-grade strictures. In **a**, all readers agreed on the presence of a high-grade stricture on the right hepatic duct (arrow). In **b**, two of the readers agreed on the absence of high-grade strictures, whereas the third reader registered a high-grade stricture in the left hepatic duct (arrowhead)

The Fleiss kappa demonstrated fair agreement between the radiologists when evaluating the presence or absence of high-grade strictures (k = 0.31) (Fig. 2, Table S2). Overall, 14% (23 out of 170) of MRCPs required consensus reading due to initial disagreement regarding the presence of high-grade strictures (Table S2). Agreement for the exact anatomical location of high-grade strictures was slight, with a Fleiss’ kappa of 0.20. The agreement for identification of extrahepatic strictures was also fair, with a kappa of 0.25. During the consensus reading session, the radiologists identified factors contributing to differences in the evaluation of strictures. Artifacts and the overall MRCP quality and resolution, precise assessment of biliary anatomy and variations, subjective perception of what defines a biliary stricture, and the precision of measurements required for high-grade strictures were described as the main reasons for disagreement.

### Assessment of high-grade and relevant strictures

**Fig. 3:**
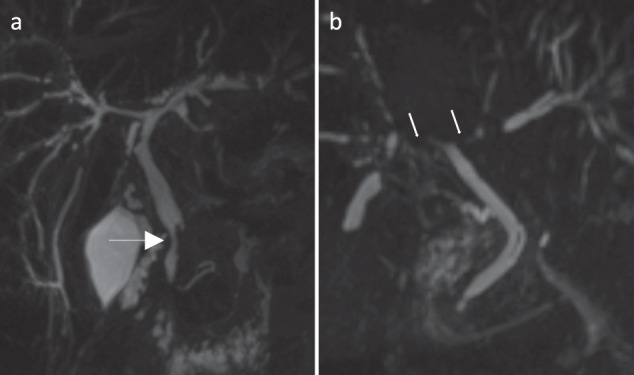
Differences in the definition of relevant strictures according to EASL and AASLD. MRCPs of two individuals with PSC. The individual in **a** had cholangitis that needed medical treatment, and the ALP level was 10.5 μkat/L (> 3 times normal). A distal common bile duct stricture was identified (arrowhead), although it was not considered high-grade (lumen narrowing < 75%). This stricture met the criteria for a relevant stricture according to AASLD but not according to EASL. The individual in **b** had cholangitis requiring treatment, and an ALP level of 12.7 μkat/L. High-grade strictures in the right and left hepatic ducts were identified (arrows), meeting the criteria for relevant strictures according to AASLD and EASL. Both individuals underwent liver transplantation due to liver decompensation

**Fig. 4:**
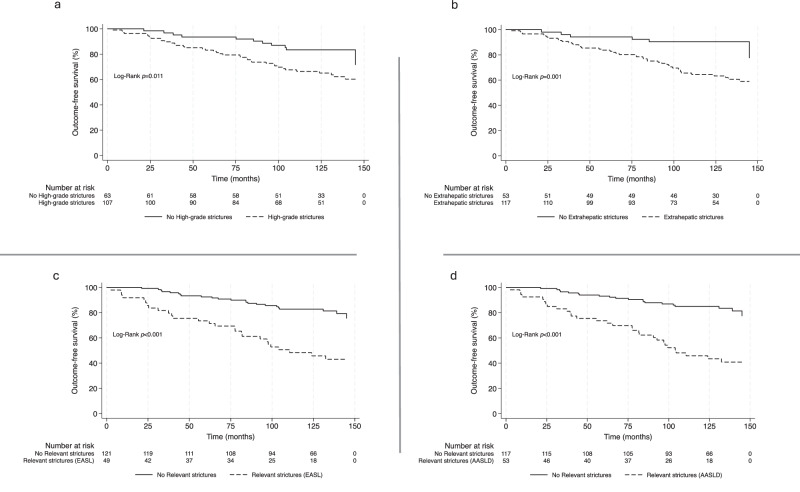
Outcome-free survival for groups of individuals with and without the various extrahepatic stricture types. **a** Outcome-free survival of individuals with and without high-grade strictures was assessed with Kaplan–Meier curves and compared with the log-rank test (*p* = 0.011). **b** Outcome-free survival of individuals with and without extrahepatic (all grades) strictures (*p* = 0.001). **c** Outcome-free survival of individuals with and without relevant strictures according to EASL (*p* < 0.001). **d** Outcome-free survival of individuals with and without relevant strictures according to AASLD (*p* < 0.001)

One hundred-seventeen individuals had extrahepatic strictures of any grade (69%), and 107 had high-grade strictures (63%). Forty-nine (29%) and 53 (31%) individuals were found to have relevant strictures according to EASL and AASLD definitions, respectively (Fig. 3). Table S4 shows the number of individuals with each of the variables present for the classification of strictures as relevant strictures according to both definitions. All 49 individuals classified as having relevant strictures according to EASL criteria also, by definition, exhibited high-grade strictures. Among those identified as having relevant strictures according to AASLD criteria, 49 had high-grade strictures (corresponding to the same 49 cases classified as relevant by EASL), while 4 did not (Table S5). All patients with relevant strictures according to EASL also met the criteria for relevant strictures according to AASLD. Thirty-nine out of 107 (36%) individuals with high-grade strictures and 44/117 (38%) individuals with extrahepatic strictures developed at least one of the outcomes. Four individuals developed CCA; three of them had high-grade strictures, and all four had extrahepatic strictures. Only one of them had relevant strictures according to both definitions. The median time between the MRCP date and CCA diagnosis was 7.8 years. Twenty-seven out of 49 (55%) individuals with relevant strictures according to EASL and 30/53 (57%) individuals with relevant strictures according to AASLD developed outcomes. Results are summarized in Table S3. Individuals with extrahepatic and high-grade strictures had worse outcome-free survival compared to those without (Fig. 4). Individuals with relevant strictures according to both definitions had worse overall survival compared to individuals who did not have relevant strictures (Fig. 4).

Table 2 shows the results from the univariate Cox proportional-hazards regression analysis, where all types of strictures were significantly associated with outcomes. In the separate multivariate models in which one type of stricture and MRS score were included, all types of strictures were significantly and independently associated with outcomes. Relevant strictures according to EASL and AASLD had HR of 2.43 (95% CI: 1.34–4.42, *p* = 0.004), and 2.89 (95% CI: 1.57–5.32, *p* = 0.001), respectively (Table 3). High-grade strictures had HR 2.00 (95% CI: 1.02–3.90, *p* = 0.044). Extrahepatic strictures were associated with outcomes with an HR of 3.34 (95% CI: 1.42–7.86, *p* = 0.006).

**Table 2:** Univariate analysis with hazard ratios (HR) evaluating the association of different types of strictures and scores with outcomes (liver transplantation, liver-related death, development of hepatobiliary malignancy)

Variable	Univariate model	
	HR (95% CI)	*p*-value
High-grade strictures	2.33 (1.19–4.55)	0.013
Extrahepatic strictures	3.82 (1.62–8.96)	0.002
Relevant strictures (EASL)	3.69 (2.12–6.45)	< 0.001
Relevant strictures (AASLD)	4.32 (2.45–7.62)	< 0.001
MELD	1.28 (1.15–1.43)	< 0.001
MRS	2.13 (1.62–2.79)	< 0.001

*MELD* model for end-stage liver disease, *MRS* Mayo risk score

**Table 3:** Multivariate models and hazard ratios (HR) evaluating the association of different types of strictures with outcomes (liver transplantation, liver-related death, development of hepatobiliary malignancy), including each time one type of stricture, and the Mayo Risk Score (MRS)

Variable	HR (95% CI)	*p*-value
High-grade strictures	2.00 (1.02–3.90)	0.044
MRS	2.14 (1.61–2.83)	< 0.001
Extrahepatic strictures	3.34 (1.42–7.86)	0.006
MRS	2.18 (1.63–2.92)	< 0.001
Relevant strictures (EASL)	2.43 (1.34–4.42)	0.004
MRS	1.86 (1.38–2.50)	< 0.001
Relevant strictures (AASLD)	2.89 (1.57–5.32)	0.001
MRS	1.81 (1.34–2.45)	< 0.001

## Discussion

In this study, we evaluated the prevalence and prognostic ability of the new definitions of high-grade and relevant strictures, which are recommended to be used in clinical practice by AASLD and EASL in individuals with large duct PSC. Our findings demonstrated that high-grade strictures were common, occurring in 63% of individuals, while relevant strictures were diagnosed in one-third. Extrahepatic strictures, high-grade strictures, and relevant strictures were all significantly associated with outcomes.

High-grade strictures were a more common finding (63%) in our study than in a previous study, where their prevalence was reported as 50% [14]. This may be attributed to both differences in patient selection and definitions. As suggested by the international liver organizations, we did not include prestenotic dilatation in the definition of high-grade strictures [6–8]. Moreover, the prevalence of high-grade strictures in our study was substantially higher (63%) than that of DS reported in a similar cohort of unselected ERCP surveillance setting (25%) [15]. Relevant strictures were present in 30% in our study, which aligns more closely with the aforementioned prevalence of DS. The term DS, based on ERCP, should be avoided in MRI reports; instead, the term high-grade stricture is recommended [6–8]. It is, however, worth mentioning that the definitions of relevant strictures/high-grade strictures and DS are fundamentally different. Therefore, the presence of relevant strictures or high-grade strictures at MRCP cannot be interpreted as a definite indicator for the presence of DS at ERCP. Correlation with ERCP findings was beyond the scope of this study, which focused exclusively on the prevalence and prognostic value of the terms high-grade and relevant strictures. Dedicated studies on the correlation between high-grade strictures at MRI, relevant strictures (according to the definitions proposed by EASL and AASLD), and DS at ERCP are an interesting and important topic for future research.

In the development study of the ANALI scores, extrahepatic strictures were not associated with radiological progression [16]. The ANALI scores were subsequently shown to be associated with outcomes in PSC, without including extrahepatic strictures as a variable [17]. However, in the present study, we showed that the presence of extrahepatic strictures is important for prognosis in PSC. Relevant strictures in PSC were introduced to better identify strictures of clinical relevance by combining information from MRCP (strictures) and clinical data, including obstructive cholestasis and bacterial cholangitis. As expected, slightly more individuals had relevant strictures according to the AASLD (31%) definition compared to the EASL (29%) definition, since the former is more inclusive and considers all types of strictures of the extrahepatic biliary tree. We found that more than half of individuals with relevant strictures (55–57%) developed outcomes, compared to 36% among those with high-grade strictures. Outcomes occurred within a median time of approximately 5 years. Despite the difference in the definition of a relevant stricture, with the AASLD definition being more inclusive, both definitions seem to have a similar prognostic ability. Having relevant strictures increased the hazard of developing outcomes about two and a half to three times (HR (EASL) = 2.43, HR (AASLD) = 2.90) in our study. Regarding the classifiers of relevant strictures, the most common was itch, followed by increased ALP. Considering the potential of high-grade and relevant strictures as prognostic biomarkers, one probable disadvantage is that both apply only to strictures of the extrahepatic biliary tree, whereas ductal changes of the intrahepatic biliary tree have been associated with clinical outcomes in PSC [17, 18]. The DiStrict score, another cholangiographic morphological imaging score, includes both intrahepatic and extrahepatic ductal changes, and has shown a strong and independent predictive ability [18]. Additionally, the DiStrict score includes dilatation of the bile ducts upstream strictures as a measure of their strength and potential to affect the liver function, and not their grade, as high-grade strictures do. The ANALI score without gadolinium also includes dilatation of the intrahepatic ducts [17]. In clinical practice, MRCP-based stricture metrics are likely to be most informative when integrated with parenchymal MRI scores and functional measurements, such as the potential functional stricture [14, 19], rather than interpreted in isolation, to provide a more comprehensive risk stratification in PSC.

Three out of four individuals with CCA had high-grade strictures, but only one of them had a relevant stricture according to the definition of both AASLD and EASL. However, CCA was diagnosed at a median time of 7.8 years after the MRI was performed. The occurrence of CCA was rare, and the high prevalence of benign high-grade strictures illustrates the challenge of diagnosing CCA and the need for additional biomarkers for CCA detection beyond the mere presence of strictures in the extrahepatic biliary tree.

We showed that the agreement for the identification of high-grade strictures was unsatisfactory, which is a crucial aspect of their clinical application. The agreement observed in our study was lower compared to the only other study on high-grade strictures [14], where the agreement was moderate. Notably, despite the readers´ long experience, the identification of any type of stricture was only fair (k = 0.25), suggesting a potential need for further standardization of stricture identification and evaluation of bile ducts with MRCP in general. Investigation of the usefulness of findings from other sequences, such as diffusion-weighted images, or from T1-w cholangiography after injection of gadoxetic acid (such as potential functional stricture (PFS) [14]), may be useful and more reproducible. Utilization of all types of clinical features, specifically radiological ones, demands robustness and reproducibility. We did not have a formal consensus training before the study, which may have influenced interreader agreement. However, these results align with several previous studies examining the interreader agreement of the evaluation of radiological findings in PSC [11, 20, 21]. The observed low agreement is a challenge that is yet to be overcome, undermining the clinical utilization of radiological findings, such as high-grade strictures and relevant strictures.

Our study has limitations. There is no well-accepted definition of cholangitis in PSC [5]. We used prospectively collected data where cholangitis was defined as cholangitis that needed medical treatment with antibiotics, which seemed to be a reasonable approach. We did not use information obtained from imaging after intravenous contrast injection to identify cholangitis, which could have contributed to a more objective definition of cholangitis. However, the goal of the study was to utilize MRCP images only. Fourteen individuals, of whom 4 developed outcomes, were excluded due to low MRCP quality. Bilirubin values were significantly higher in the excluded patients; nevertheless, within the normal range of values, suggesting slightly more advanced disease. However, all other laboratory parameters, including MELD score, albumin, and ALP, were not statistically different. Furthermore, we focused on assessing the interreader agreement and did not assess the intrareader agreement. Despite the relatively large sample size of our study population (*n* = 170), the number of individuals without high-grade strictures was somehow small, due to the high prevalence of high-grade strictures in our cohort, which may have affected our results. Moreover, we did not assess the longitudinal changes of strictures, namely whether strictures developed between exams, which may impact the potential usefulness of these strictures and warrant further evaluation in future studies. Lastly, although major hardware and software upgrades did not occur during the study period and MRCP parameters were kept stable, minor protocol variations over time cannot be completely excluded.

We conclude that extrahepatic and high-grade strictures are common in PSC, yet their identification and consequently identification of relevant strictures seem to be challenging even for experienced radiologists. The presence of all types of extrahepatic strictures, including those classified as relevant by either EASL or AASLD definition, seems to be associated with a poorer prognosis. However, the insufficient agreement for their identification limits the potential of direct clinical implementation. These findings, nevertheless, encourage the simultaneous utilization of radiological and clinical features in the pursuit of good surrogate endpoints for clinical trials; however, larger multicenter studies are needed.

## Supplementary information

ELECTRONIC SUPPLEMENTARY MATERIAL

## Abbreviations

AASLD: American Association for the Study of Liver Diseases
CCA: Cholangiocarcinoma
DS: Dominant strictures
EASL: European Association for the Study of the Liver
ERCP: Endoscopic retrograde cholangiopancreatography
HR: Hazard ratio
IPSCSG: International PSC study group
MELD: Model for end-stage liver disease
MRS: Mayo risk score
PSC: Primary sclerosing cholangitis
