# Supplementary material for: Prevalence, interreader agreement, and prognostic value of high-grade and relevant strictures in individuals with primary sclerosing cholangitis
Source: Eur Radiol. 2026 Mar 13;36(7):5787–95. doi: 10.1007/s00330-026-12426-6 (PMC13282313; doi:10.1007/s00330-026-12426-6)
Supplement: Supplementary file 1 — ELECTRONIC SUPPLEMENTARY MATERIAL [file 330_2026_12426_MOESM1_ESM.pdf]

Prevalence, interreader agreement, and prognostic value of high-grade and relevant strictures in individuals with primary sclerosing cholangitis

ELECTRONIC SUPPLEMENTARY MATERIAL

Eur Radiol (2026) Hamma S, Bergquist A, Andersson M, Villard C, Grigoriadis A.

Table of contents

Table S1 3

Table S2 4

Table S3. 5

Table S4 6

Table S5 7

Table S6 8

Figure S1 9

Table S1: Sequences of the standardized protocol used in the study.

Sequence	Plane	Thickness (mm)	TE (ms)	TR (ms)
**T2-w HASTE**	Axial	4	124	1500
**T2-w HASTE**	Coronal	4	76	1080
**T1-w 2D GRE in/opposed**	Axial	4	5.05/2.37	126
**T2-w 3D SPACE MRCP**	Axial	2.5	685	4536
**T2-w 3D SPACE MRCP**	Coronal	1	685	3832
**T1-w VIBE before contrast**	Axial	2.5	1.92	4.29
**T1-w VIBE post contrast[a]**	Axial	2.5	1.92	4.29
**DWI[1,b]**	Axial	5	77	5000

[1]DWI; Diffusion weighted imaging

[a]0.1 ml/kg body weight of gadoxetic acid (Primovist, Bayer Healthcare); [b]b-values: 50,400, 800 s/mm^2

Table S2. Number of cases and percentage (in parentheses) of disagreement between readers for the presence of high-grade strictures and strictures of any grade.

Parameter	Cases of disagreement between readers
**High-grade strictures**	23 (14%)
**Strictures of any grade**	24 (14%)

Table S3. **Number and percentages in parentheses of individuals with high-grade strictures (HGS), extrahepatic strictures, relevant strictures according to the definitions of EASL (RS EASL) and AASLD (RS AASLD), with and without outcomes, along with time to outcome development, and individuals with signs or symptoms of cholangitis according to the study definition, in the study population.**

**Variable**	**Number**	
	**n (%)**	**N total**
**HGS**	107 (63)	170
**Extrahepatic strictures**	117 (69)	170
**HGS + outcome**	39 (36)	107
**Extrahepatic strictures + outcome**	44 (38)	117
**RS EASL**	49 (29)	170
**RS AASLD**	53 (31)	170
**RS EASL + outcome**	27 (55)	49
**RS EASL Median time to outcome in months (range)**	62 (2,6-132,4)	49
**RS AASLD + outcome**	30 (57)	53
**RS AASLD Median time to outcome in months (range)**	62,8 (2,6-132,4)	53
**Signs / symptoms of cholangitis**	70 (41)	170

Table S4. Number of patients with high bilirubin, high ALP, itch, and bacterial cholangitis that were described as having relevant strictures according to EASL (RS EASL) and AASLD (RS AASLD) definitions.

**Variable**	**Parameter**					
	**n**	**HGS**	**High bilirubin**	**High ALP**	**Itch**	**Cholangitis**
**RS EASL**	49	49	2	19	35	10
**RS AASLD**	53	49	2	20	37	11

HGS; High-grade strictures, High bilirubin>50 μmol/l, High ALP > 1.5 UNL

Table S5. Number of patients with and without high-grade strictures (HGS), and relevant strictures according to EASL (RS EASL) and AASLD (RS AASLD) definitions.

Relevant strictures	High-grade strictures		
	n	HGS	No HGS
RS EASL	49	49	0
RS AASLD	53	49	4

Table S6. Comparison between laboratory values and demographics between the final study population and excluded patients due to low image quality. Categorical/ordinal variables were compared with Fisher´s exact test, whereas continuous variables were compared with the Mann-Whitney test. For continuous variables, the values are presented in medians. Statistically significant values are marked with an asterisk.

Variable	Study population (n=170)	Excluded (n=14)	*p*-value
**Sex (male/female)**	106/64	12/2	0.090
**Age at MRI**	40	40	0.483
**Bilirubin**	11	19	0.019*
**ALAT**	0.80	0.73	0.920
**ASAT**	0.67	0.68	0.718
**GT**	2.2	1.2	0.585
**ALP**	2.1	2.5	0.916
**ALB**	39	37	0.512
**CA 19-9**	9.4	17.5	0.051
**MELD**	6	8	0.093
**IBD presence/type**	-	-	0.389
**Outcome**	50	4	0.607
**Cholangitis**	70	4	0.607

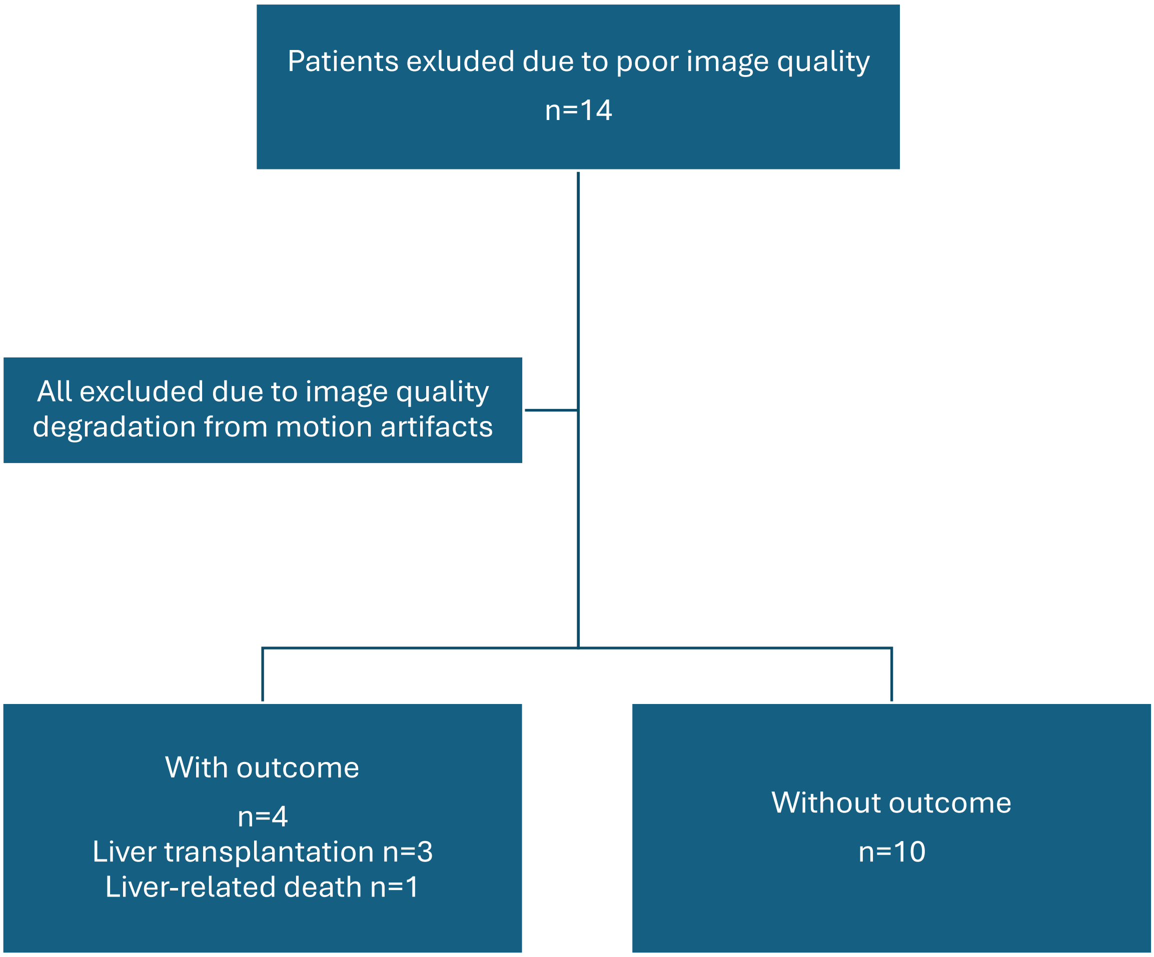

Figure S1: Flow chart of excluded patients, reasons for exclusion, and type of outcome that occurred, if any.
